# Prospective Placebo-Controlled Assessment of Spore-Based Probiotic Supplementation on Sebum Production, Skin Barrier Function, and Acne

**DOI:** 10.3390/jcm12030895

**Published:** 2023-01-23

**Authors:** Iryna Rybak, Kelly N. Haas, Simran K. Dhaliwal, Waqas A. Burney, Aunna Pourang, Simran S. Sandhu, Jessica Maloh, John W. Newman, Robert Crawford, Raja K. Sivamani

**Affiliations:** 1 Department of Dermatology, University of California—Davis, Sacramento, CA 95816, USA; 2 Department of Biology, University of Massachusetts, Amherst, MA 01003, USA; 3 Integrative Skin Science and Research, Sacramento, CA 95815, USA; 4 Department of Dermatology, Wayne StateUniversity, Dearborn, MI 48124, USA; 5 School of Medicine, University of California—Davis, Sacramento, CA 95816, USA; 6 Department of Nutrition, University of California—Davis, Sacramento, CA 95616, USA; 7 West Coast Metabolomics Center, Genome Center, Davis, CA 95616, USA; 8 United States Department of Agriculture, Agricultural Research Service, Western Human Nutrition Research Center, Davis, CA 95616, USA; 9 Department of Biological Sciences, California State University, Sacramento, CA 95819, USA; 10 College of Medicine, California Northstate University, Elk Grove, CA 95757, USA; 11 Pacific Skin Institute, Sacramento, CA 95815, USA

**Keywords:** probiotics, short-chain fatty acids, gut microbiome, acne, sebum, TEWL

## Abstract

Probiotic supplementation has been shown to modulate the gut–skin axis. The goal of this study was to investigate whether oral spore-based probiotic ingestion modulates the gut microbiome, plasma short-chain fatty acids (SCFAs), and skin biophysical properties. This was a single-blinded, 8-week study (NCT03605108) in which 25 participants, 7 with noncystic acne, were assigned to take placebo capsules for the first 4 weeks, followed by 4 weeks of probiotic supplementation. Blood and stool collection, facial photography, sebum production, transepidermal water loss (TEWL), skin hydration measurements, and acne assessments were performed at baseline, 4, and 8 weeks. Probiotic supplementation resulted in a decreasing trend for the facial sebum excretion rate and increased TEWL overall. Subanalysis of the participants with acne showed improvement in total, noninflammatory, and inflammatory lesion counts, along with improvements in markers of gut permeability. The gut microbiome of the nonacne population had an increase in the relative abundance of *Akkermansia*, while the subpopulation of those with acne had an increase in the relative abundance of *Lachnospiraceae* and *Ruminococcus gnavus*. Probiotic supplementation augmented the circulating acetate/propionate ratio. There is preliminary evidence for the use of spore-based probiotic supplementation to shift the gut microbiome and augment short-chain fatty acids in those with and without acne. Further spore-based supplementation studies in those with noncystic acne are warranted.

## 1. Introduction

The use of antibiotic treatment courses is common for several inflammatory dermatology conditions, such as acne vulgaris, rosacea, and hidradenitis suppurativa. The chronic use of antibiotics increases the development of drug-resistant bacteria, such as *Cutibacterium acnes* or *Staphylococcus aureus* [1,2]. Apart from drug-resistant bacteria, long-term antibiotic use can lead to gut dysfunction and the emergence of antibiotic-resistant gut bacteria [3].

There is growing interest in understanding how the gut microbiome may communicate with the skin. Specifically, gut bacteria are thought to engage different modes of communication with the skin and to comprise the gut–skin axis [4]. Previous investigations have reported that the gut microbiome may influence the immune system and inflammation in conditions such as psoriasis [5,6,7], acne [8,9], rosacea [10], and atopic dermatitis [11]. Accordingly, there is more interest in understanding how oral probiotics may modulate the skin. Probiotics have been shown to exhibit anti-inflammatory effects locally and distally from the gut [12,13,14].

Several studies indicate that oral probiotic intake may be beneficial to the skin. For example, probiotics may improve atopic dermatitis [15]. A probiotic consisting of *Lactobacillus acidophilus*, *Lactobacillus delbrueckii* subspecies *bulgaricus*, and *Bifidobacterium bifidum* was shown to be similarly effective to minocycline 100 mg daily for acne [16]. Oral supplementation with *Bifidobacterium breve* can attenuate UV-induced decreases in skin hydration and increases in transepidermal water loss (TEWL) [17,18]. Another study with ingestion of *B. breve* fermented milk improved skin hydration in human volunteers [19].

Despite this emerging evidence, it still remains unclear how probiotics are mechanistically communicating with the skin and how they may globally affect the skin’s biomechanical properties, such as sebum production, transepidermal water loss, and skin hydration. Although fermented foods have been shown to increase skin hydration [19], human studies that assess how probiotic supplementation influences sebum production and skin barrier properties are sparse.

The purpose of this study was to prospectively evaluate how oral spore-based probiotics may alter skin biomechanical properties and sebum production. In particular, acne is a multifactorial disease that is driven by the hyperproliferation of epidermal keratinocytes, sebum production by sebocytes, inflammation that is believed to be secondary to the overgrowth of *Cutibacterium acnes*, and scarring that results from ongoing inflammation [20,21,22]. Accordingly, we included the recruitment of participants with noncystic acne to further stratify the population. Finally, we assessed changes in the gut microbiome and plasma short-chain fatty acids to correlate these factors to changes in the skin.

## 2. Materials and Methods

### 2.1. Study Participants

This 8-week single-blinded, placebo-controlled study was conducted from June to October 2018. The Institutional Review Board at the University of California, Davis, approved this study (Protocol #1242039), and it was registered on ClinicalTrials.gov (NCT03605108). All participants provided written informed consent prior to participation and received financial compensation. Twenty-five healthy participants (mean age: 30.8 years; range: 19–62 years) were screened and enrolled through the UC Davis Dermatology clinic. Clinical research coordinators enrolled and assigned the interventions. Exclusion criteria included any topical antibiotic or benzoyl-peroxide-containing product within one month of participation, individuals with BMI higher than 30 kg/m^2^, subjects that started a new hormonal birth control agent or switched to a different hormonal birth control within the previous two months, individuals who had used a systemic antibiotic or systemic antifungal within 1 month of study participation, individuals who were exposed to oral isotretinoin, individuals who were using or had used a topical retinoid during the previous 14 days, and subjects who were using medications that alter blood lipids such as statins and antihyperlipidemic medications. Participants with cystic acne were excluded, but subjects with noncystic acne (*n* = 7) were eligible. Finally, subjects were excluded if they were current smokers, had smoked tobacco over the past year, or had a 5 pack/year smoking history. Participants were allowed to use their current facial regimen so long as the regimen was not changed during the study. Participants were directed to avoid any facial cosmetic procedures while participating.

### 2.2. Study Design and Intervention

**Figure 1:**
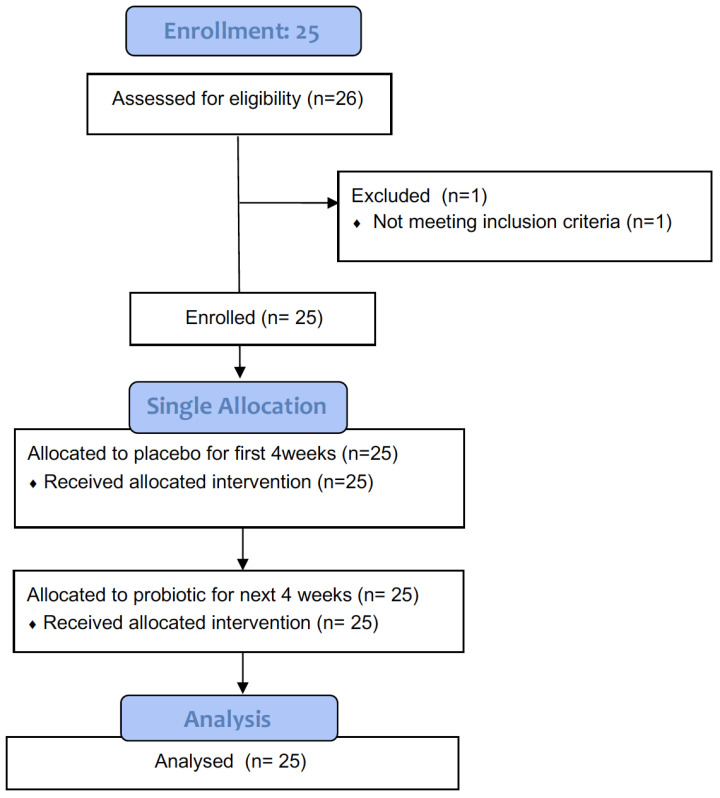
CONSORT diagram for the clinical study.

The study consisted of three visits (baseline, week 4, and week 8), as shown in Figure 1. Twenty-five subjects received placebo pills for the first four weeks, and the same twenty-five subjects then received spore-based probiotics (Megasporebiotic, Microbiome Labs, Saint Augustine, FL) consisting of 4 billion spores from Gram-positive, spore-forming strains (*Bacillus indicus* (HU36), *Bacillus subtilis* (HU58), *Bacillus coagulans*, *Bacillus licheniformis*, and *Bacillus clausii*) for the next four weeks. Subjects were instructed to take two capsules per day. Subjects were instructed not to wash their faces or body or apply any products to their faces on the day of their study visit.

### 2.3. Blood Collection

Fasting blood samples were collected at baseline, week 4, and week 8 to assess baseline and endpoint values of short-chain fatty acids and cytokines related to inflammation and intestinal barrier permeability (TNFa, LPS, FABP-2, zonulin) to correlate this with the microbiome assessments.

### 2.4. Cytokine Measurement ELISA

Blood collected from participants was centrifuged at 1200 rpm at room temperature for 10 min, and then plasma was collected in methanol-washed Eppendorf tubes, aliquoted, and stored in a −80 °C freezer. Freeze–thaw cycles were avoided, and each sample was thawed only once prior to use. ELISA assays were performed according to the manufacturer’s protocol for FABP2 and TNFa (Thermo Fisher Scientific, Frederick, MD, USA) and LPS and zonulin (MyBioSource, San Diego, CA, USA). All cytokine measurements were performed in triplicate.

### 2.5. Facial Photography

High-resolution photographs were taken at baseline, week 4, and week 8 with the use of the BTBP 3D Clarity Pro Facial Modeling and Analysis System (Brigh-Tex BioPhotonics, San Jose, CA, USA). The photographic instrumentation takes automated photographs in zero extraneous ambient lighting with reproducible placement of the face and identical photographic exposures. This system has been validated in comparison to clinical grading of multiple facial features [23,24,25,26].

### 2.6. Facial Grading and Analysis

Photographic grading was performed by a board-certified dermatologist in a blinded fashion. Subjects with acne were assessed for changes in the acne by quantifying the inflammatory and noninflammatory lesions.

### 2.7. Skin Barrier Function

The investigators assessed skin barrier function by measuring sebum production (Sebumeter SM 815; Courage and Khazaka, Cologne, Germany), transepidermal water loss (TEWL, Vapometer; Delfin Technologies, Stamford, CT, USA), and hydration (MoistureMeterSC; Delfin Technologies, Stamford, CT, USA) at baseline, 4, and 8 weeks.

### 2.8. Skin Microbiome Collection

Nasolabial and glabellar skin was swabbed under sterile conditions with sterile swabs to collect specimens for microbiome analysis at baseline, 4, and 8 weeks. Copan-e swabs (480C) were used to collect microbiome samples. Swabs were collected into 300 µL Copan-e buffer in sterile DNase-free microfuge tubes in order to minimize sample volume. Swabs were stored at −80 °C until DNA was extracted. At each visit, a noninvasive adhesive pore cleansing strip (Biore, Cincinnati, OH, USA) was applied along with sebutapes (Cuderm, Dallas, TX, USA).

### 2.9. Gut Microbiome Collection

Fecal stool samples were collected to determine how the gut microbiome and lipidome shifts at baseline, 4, and 8 weeks. Subjects were given at-home stool collection kits. Kits were expected to be used within 24 h of the weeks 0, 4, and 8 visits. The subjects were instructed to keep the stool in their at-home freezer until their clinical visit. Stool collection kits included ice packs to keep the sample cool upon transport. Once received, stool samples were kept at −80 °C until processing.

### 2.10. Microbiome Analysis

Fecal samples were defrosted on ice, and sterile spatulas were used to transfer 0.25 ± 0.05 g fecal material into Qiagen PowerSoil (12888-100) bead tubes. After bead-beating, samples were sterilized by heating at 80 °C for 3 min, frozen, then thawed, and treated with 10 mg/mL lysozyme for 2 h at 42 °C. DNA was eluted in 60 µL instead of 100 µL. Skin swabs were processed in the same manner, except that heat killing and lysozyme steps were performed prior to bead-beating.

The V3-V4 region of the *16S rRNA* gene was amplified for sequencing. The V3 F primer was the same for both fecal and skin samples:

5′-TCGTCGGCAGCGTCAGATGTGTATAAGAGACAG.

For the fecal samples, previously published V4 R primers were used [27]. V4 F skin microbiome-specific primer was:

5′-GTCTCGTGGGCTCGGAGATGTGTATAAGAGACAG.

Phusion high-fidelity DNA polymerase (ThermoFisher F530L) was used for library preparation: 1 U polymerase, 2 mM MgCl_2_, 5% DMSO, 0.5 µM each primer, 0.2 mM dNTPs in a final reaction volume of 25 µL. A total of 3 µL fecal DNA or 5 µL swab DNA was used per reaction. For reactions that did not result in bands, PCR was retried with more DNA (5 µL for fecal and 10 µL for swab).

The PCR program included a 2 min hot start (98 °C), followed by 30 cycles of 98 °C for 30 s, 62 °C for 30 s, and 72 °C for 15 s, with a final extension at 72 °C for 30 s before pausing at 4 °C. All samples were run on a gel to ensure the PCR was successful and quantified using the Qubit Fluorometer system. Samples were sent to the University of California Berkeley for barcoding and sequencing. A 300-cycle paired-end sequencing was performed on the Illumina MiSeq platform (Illumina, San Diego, CA USA).

Sequencing data were processed in Qiime2 [28]. A variety of PCoA plots were constructed, Shannon diversity was calculated and compared by group and treatment, and *t*-tests, fold-changes, and Δ relative abundances were calculated for each taxon and compared across groups/treatments. Trends were also visualized, and variances were calculated per taxon. For abundant operational taxonomic units (OTUs) not well-resolved using the Qiime2 classifier, phylogenies were constructed to better place them and to further parse or clump OTUs. Type sequences were pulled from the Ribosomal Database Project (RDP) website [29]; MEGA7 was used to align sequences and construct phylogenies [30].

### 2.11. Short-Chain Fatty Acid Quantification

Plasma SCFAs were isolated and quantified as dimethyl-tert-butylsilane (DiMTBS) derivatives by GC-MS. Specifically, plasma samples (250 µL) isolated from EDTA containing sampling tubes and aqueous calibration solutions, and procedural LC-MS water blanks were enriched with 5 µL of 5.24 mM d3-acetate and 0.259 mM d5-propionate (CDN), acidified with 15 µL 6 N hydrochloric acid, and extracted with 1 mL of MTBE. Samples were centrifuged for 5 min at 10,000 rcf, and 0.5 mL of the supernatant was dried with ~100 mg of sodium sulfate for 10 min. A 100 µL subaliquot was incubated with 15 µL of MTBSTFA +1% TBDMS (Sigma-Aldrich, St. Louis, MO, USA) at 50 °C for 90 min and allowed to sit at room temperature overnight. Samples were then enriched with 10 µL of 272 µM 15:1n5 methyl ester internal standard. Residues were separated on 6890 GC equipped with a 30 m × 0.25 mm, 0.25µm DB-5 ms, and 5973N MSD (Agilent Technologies, Santa Clara, CA, USA) using a 1:10 split of a 2 µL injection, electron impact ionization, and selected ion monitoring/full scan mass spectra generation. GC parameters: injection port—280 °C; oven program—100 °C (hold 2 min), 35 °C/min to 280 °C; carrier gas—1.5 mL/min helium; total flow—19 mL/min. Data were acquired and processed with MassHunter v B.08. Acetate was corrected for d3-acetate recoveries, while propionate and butyrate were corrected for d5-propionate recoveries.

### 2.12. Statistical Analysis

The primary outcome measures were to assess whether probiotics could reduce sebum production after 4 weeks of probiotic supplementation. Secondary outcome measures included shifts in the gut microbiome, changes in skin barrier biophysical properties, skin microbiome changes, and changes in blood SCFAs. The study participants were analyzed as an overall group and then subdivided into two groups, Acne and No Acne. The data were analyzed at baseline, 4, and 8 weeks. The alpha was set to 0.05, and a repeated measure Wilcoxon test was used to perform statistical analysis. In addition, *p*-values were considered significant if they were less than 0.05 and are reported as approaching significance if between 0.05 and 0.2. Prism v.9 (GraphPad Software LLC, San Diego, CA, USA) was utilized to analyze the data.

## 3. Results

### 3.1. Skin Biophysical Properties

**Figure 2:**
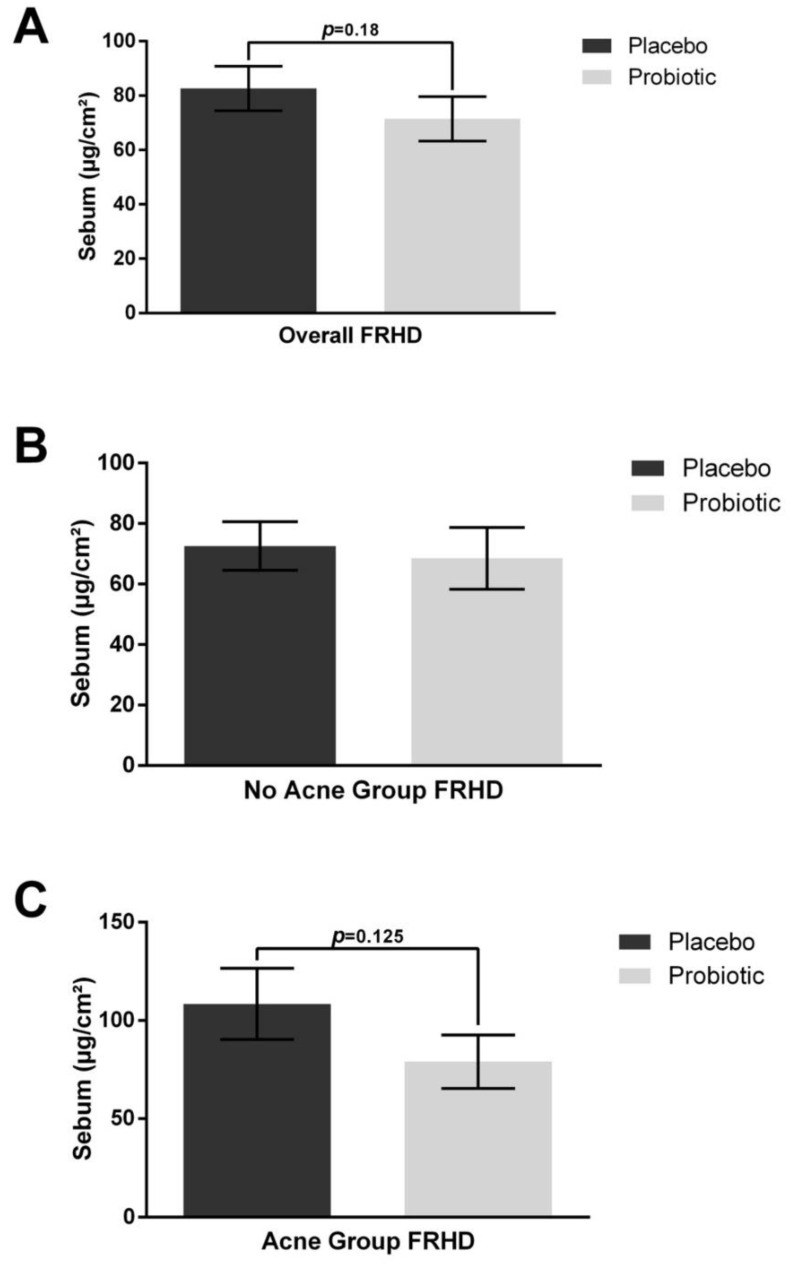
Sebum excretion rate. Forehead (FRHD) measurements are depicted for the overall population as well as those without and with acne. (**A**) Overall, sebum excretion had a decreasing trend after probiotic ingestion. (**B**) Sebum excretion was unchanged with placebo or probiotic ingestion in the no acne group (*n* = 18). (**C**) A decreasing trend for sebum excretion was noted after probiotic exposure in those with acne (*n* = 7, *p* = 0.125), whereas there was no change in the placebo treatment period. Error bars represent mean + SEM.

Changes in the skin barrier and the sebum production rate were assessed. The placebo intervention induced no change in the sebum excretion rate, but the probiotic intervention had a decreasing trend in the sebum excretion rate by 13% (Figure 2, *p* = 0.18). The sebum excretion rate remained unchanged with placebo or probiotic exposure after stratifying for those without acne. However, when stratifying for those with acne, there was a marked 28% decrease in the sebum excretion rate after probiotic exposure that approached significance (Figure 2, *p* = 0.125), while there was no change during the placebo intervention.

**Figure 3:**
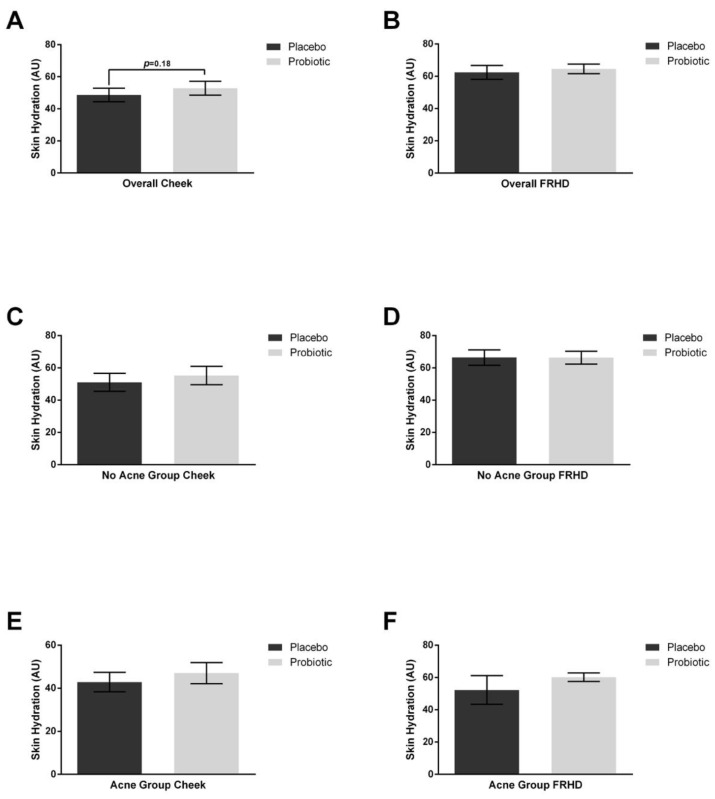
Skin hydration. After supplementation with 4 weeks of placebo and 4 weeks of probiotics, skin hydration was measured on the cheek and forehead. (**A**) Skin hydration trended toward an increase (*p* = 0.18) after probiotic supplementation on the cheeks, but there was no difference noted on the forehead (**B**). Subanalysis in those without acne did not show any differences on the cheeks (**C**) or forehead (**D**). Subanalysis among those in the acne group did not show any differences on the cheek (**E**) or the forehead (**F**). Error bars represent mean + SEM.

Skin hydration on the cheek had an increasing trend with probiotic supplementation (Figure 3, *p* = 0.18) but not with placebo. Forehead hydration was not found to have any differences.

**Figure 4:**
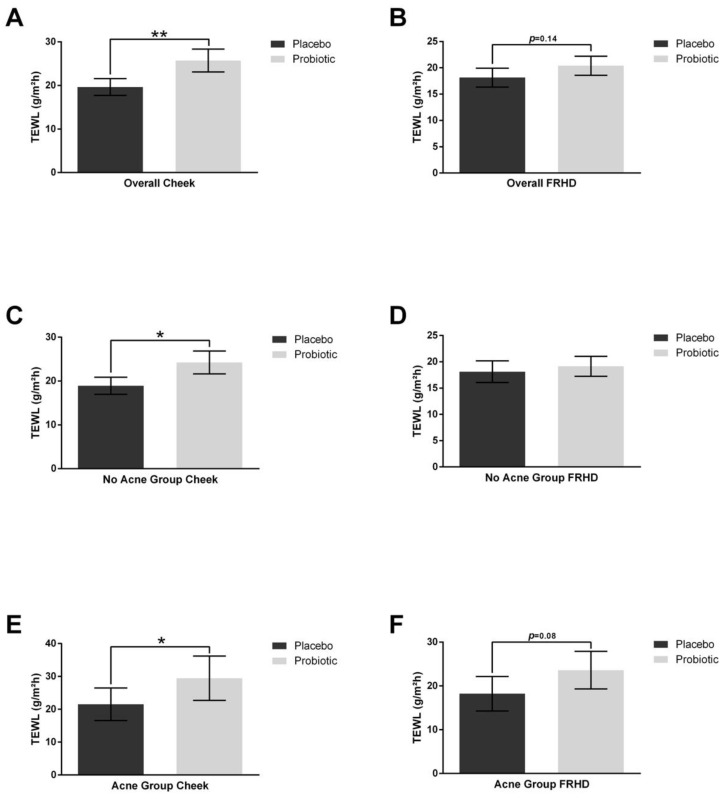
Skin transepidermal water loss (TEWL). TEWL was measured after placebo and probiotics supplementation for 4 weeks. Probiotic treatment increased TEWL on the cheek (**A**) and trended up on the forehead (**B**). In the no acne group, probiotic supplementation increased TEWL on the cheek (**C**) but not on the forehead (**D**). In the group with acne, probiotic supplementation increased the TEWL on the cheek (**E**) and the forehead (**F**). Error bars represent mean + SEM, * *p* ≤ 0.05, ** *p* ≤ 0.01.

After probiotic supplementation, cheek transepidermal water loss (TEWL) was increased overall and in those with and without acne (Figure 4). Similarly, the forehead had an increasing trend in the overall population (*p* = 0.14) and in those with acne (*p* = 0.08).

### 3.2. Gut-Derived Proteins and TNF-Alpha

**Figure 5:**
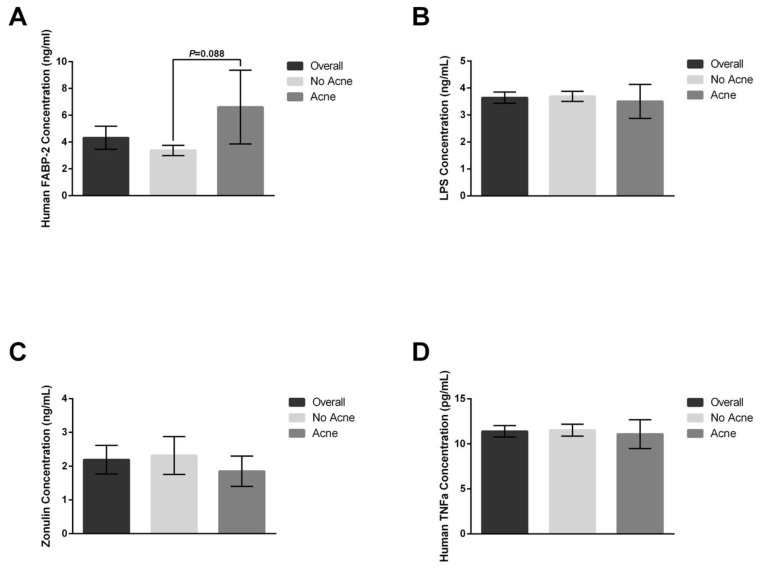
Baseline plasma markers for “leaky gut” and inflammation. Plasma concentrations were measured after participants were divided into two groups based on the presence or absence of acne. (**A**) Mean human FABP-2 concentration had an increasing trend within the acne group compared to the no acne group (*p* = 0.088). (**B**) Mean overall lipopolysaccharides (LPS), zonulin (**C**), or TNF-alpha (**D**) concentrations were different among the groups. Error bars represent mean + SEM.

Several blood markers offer insight into inflammation and intestinal permeability (“leaky gut”), and we sought to understand how probiotic interventions may impact these markers. At baseline, there was no difference between those with and without acne for TNF-alpha, zonulin, or LPS. However, FABP-2 levels were elevated in those with acne relative to those without acne (Figure 5, *p* = 0.088) prior to any interventions.

**Figure 6:**
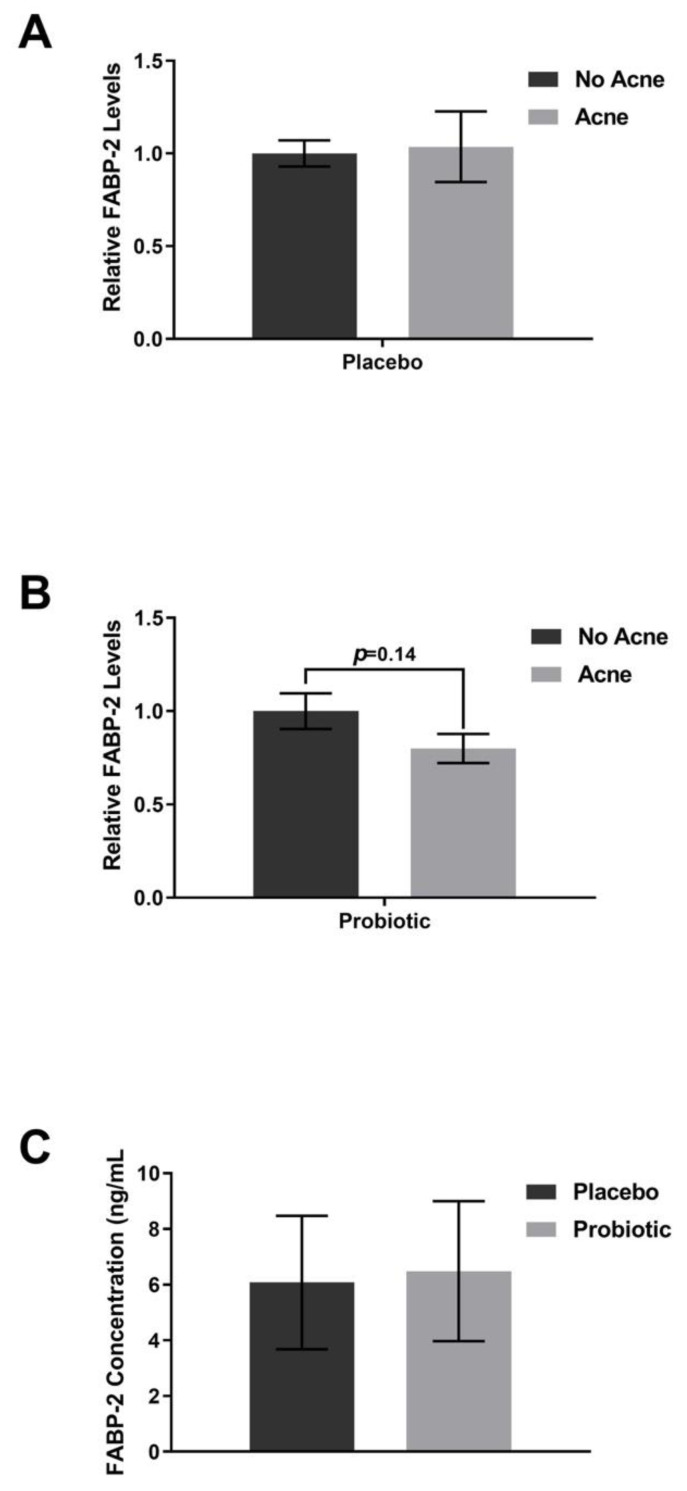
FABP-2 levels. Participants received placebo for the first 4 weeks and probiotics for the next 4 weeks. (**A**) Placebo supplementation did not lead to differences between the acne and no acne groups. (**B**) Probiotic exposure led to a decreasing trend in FABP-2 in the acne group compared with the no acne group. (**C**) There was no significant change after probiotic exposure within the acne subpopulation. Error bars represent mean + SEM.

**Figure 7:**
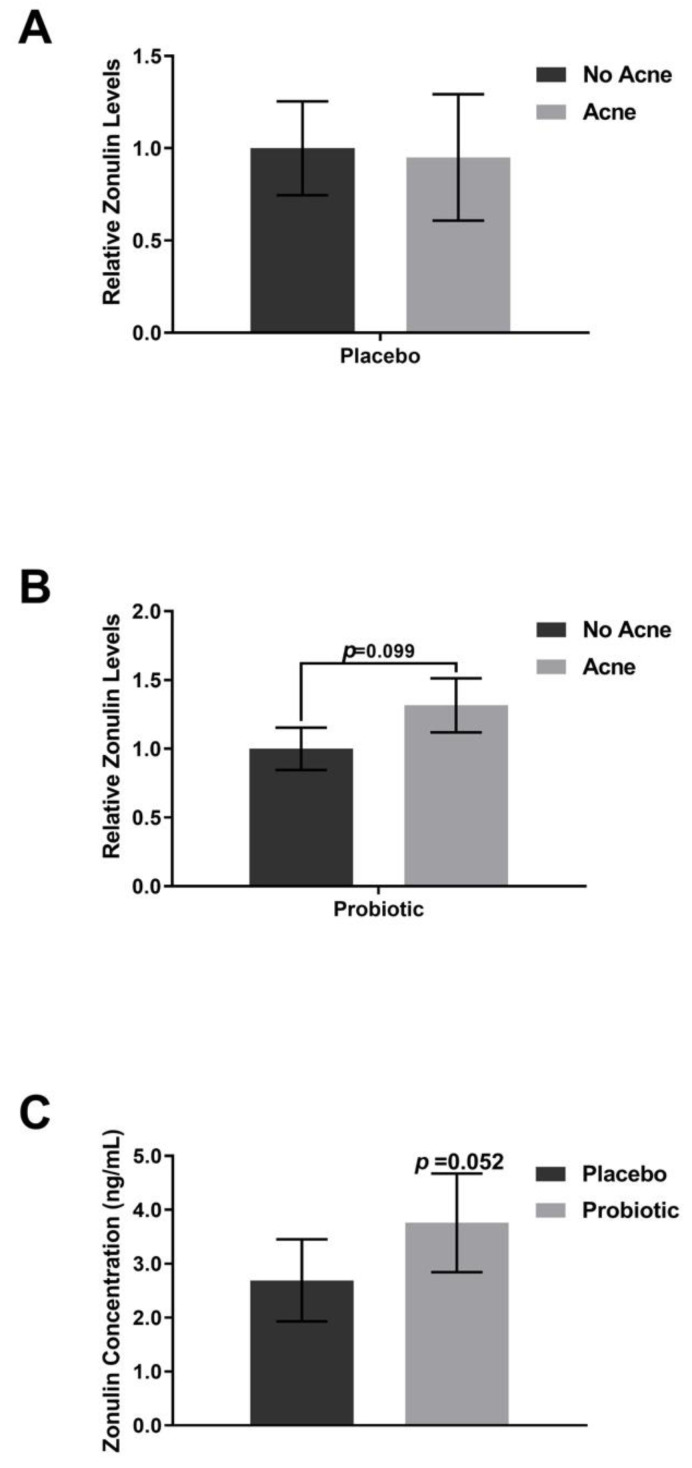
Zonulin levels. Participants received placebo for the first 4 weeks and probiotics for the next 4 weeks. (**A**) Placebo supplementation did not lead to differences between the acne and no acne groups. (**B**) Probiotic exposure led to an increasing trend in zonulin in the acne group compared with the no acne group. (**C**) There was a trend toward an increase within the acne subpopulation. Error bars represent mean + SEM.

**Figure 8:**
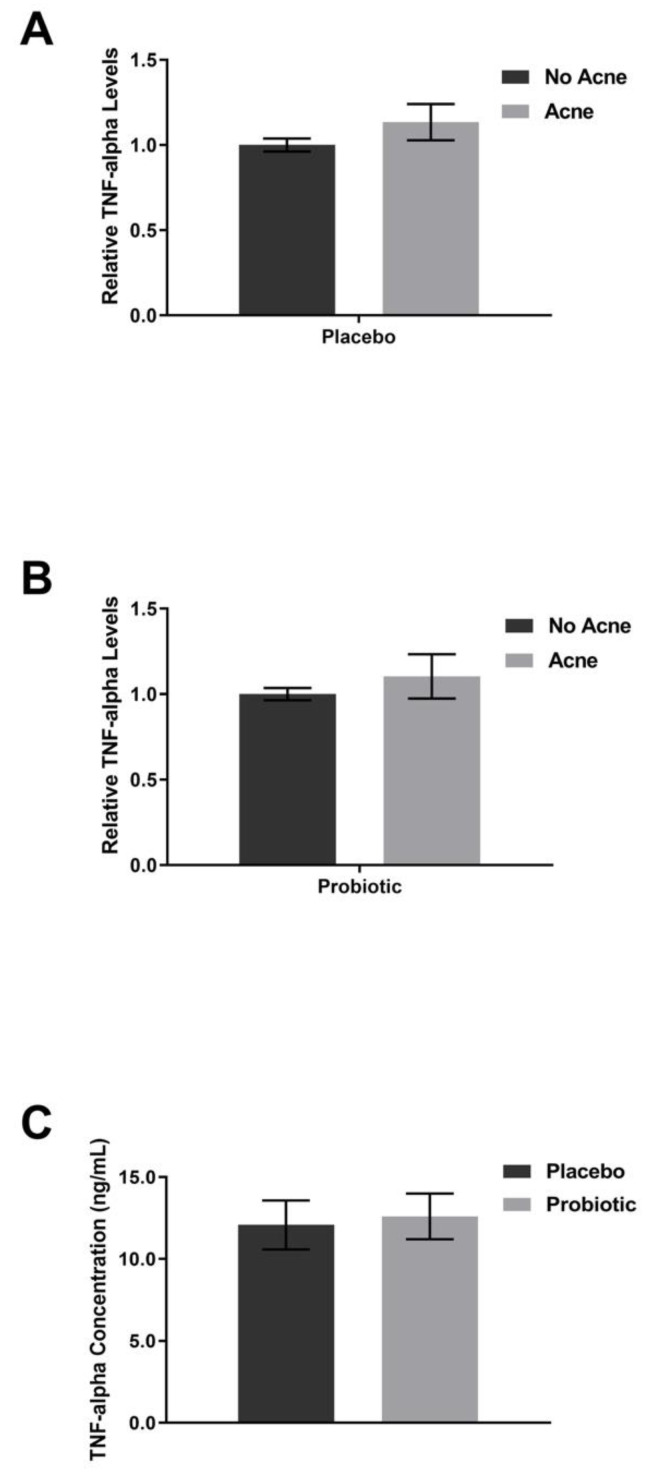
TNF-alpha levels. Participants received placebo for the first 4 weeks and probiotics for the next 4 weeks. (**A**) Placebo supplementation did not lead to differences between the acne and no acne groups. (**B**) Probiotic exposure did not lead to differences in the acne group compared with the no acne group. (**C**) There was no significant change after probiotic exposure within the acne subpopulation. Error bars represent mean + SEM.

**Figure 9:**
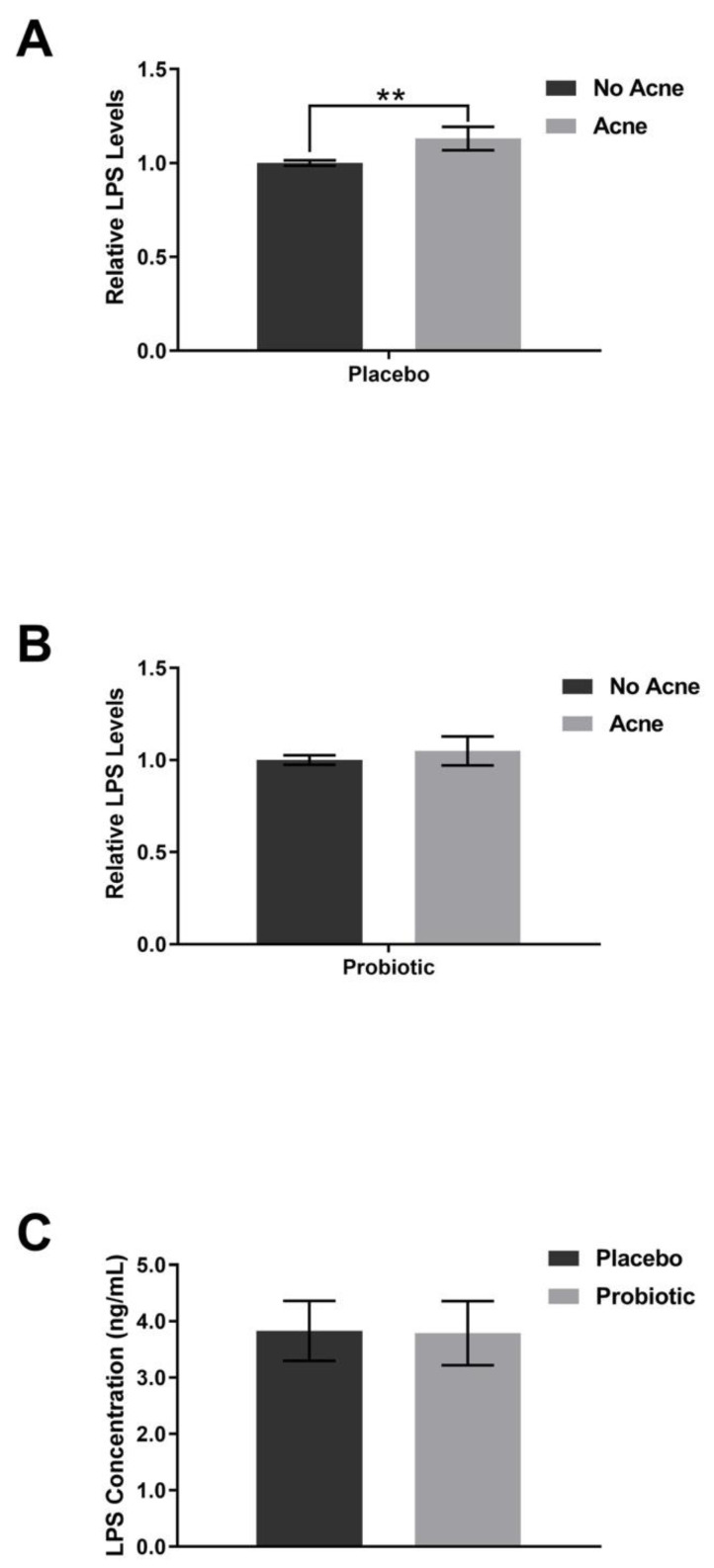
LPS levels. Participants received placebo for the first 4 weeks and probiotics for the next 4 weeks. (**A**) Placebo supplementation did not lead to differences between the acne and no acne groups. (**B**) Probiotic exposure did not lead to differences in the acne group compared to the no acne group. (**C**) There was no significant change after probiotic exposure within the acne subpopulation. Error bars represent mean + SEM, ** *p* ≤ 0.05.

Supplementation with the placebo did not shift the plasma levels of FABP-2 (Figure 6), zonulin (Figure 7), or TNF-alpha (Figure 8). The lipopolysaccharides (LPSs) level increased by 9.2% in those with acne after placebo exposure but not in the group without acne (Figure 9, *p* < 0.05). Exposure to the probiotic led to a normalization of LPS (Figure 9), while the levels of zonulin and TNF-alpha remained unchanged. When comparing the acne group against the no acne group, there was a trend toward a decrease in FABP-2 (*p* =0.14) and a trend toward an increase in zonulin (*p* = 0.099) after probiotic exposure. When the data were evaluated for shifts from probiotic exposure in those with acne, there were no significant shifts in FABP-2 (Figure 6C), TNF-alpha (Figure 8C), or LPS (Figure 9C), but there was a trend toward an increase in zonulin (Figure 7C, *p* = 0.052).

### 3.3. Acne Response to Probiotic Intervention

**Figure 10:**
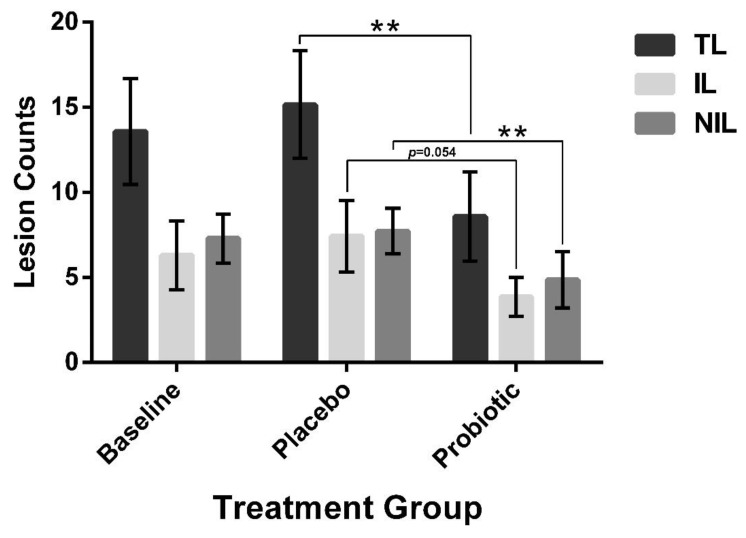
Acne lesion counting. Acne lesions were counted at each visit in participants with noncystic acne (*n* = 7). Placebo supplementation for 4 weeks did not lead to any change in the lesion counts. However, the total lesion (TL) and noninflammatory lesion (NIL) count decreased with 4 weeks of probiotic supplementation. The inflammatory lesion (IL) decreased and approached significance (*p* = 0.054). Error bars represent mean + SEM, ** *p* ≤ 0.01.

Acne was graded by lesion counting. Placebo supplementation did not lead to any significant changes in the lesion counts (Figure 10). However, probiotic supplementation significantly decreased the noninflammatory lesions and the total lesion counts, while the decrease in inflammatory lesions approached significance (*p* = 0.054).

### 3.4. Changes in the Gut and Skin Microbiome

**Figure 11:**
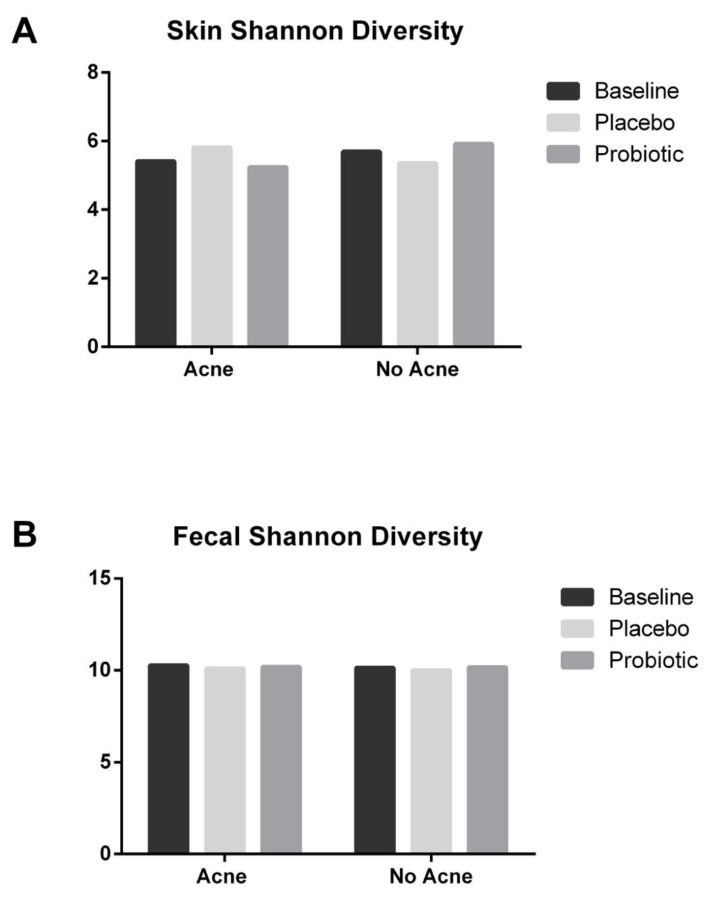
Shannon diversity of microbiome. Skin and gut microbiome Shannon diversities did not change with probiotic supplementation in either the acne or the no acne group. (**A**) Skin Shannon diversity; (**B**) Fecal Shannon diversity.

Gut and skin microbiome diversity was assessed by the Shannon diversity. Neither the skin nor the gut microbiome Shannon diversity significantly changed after the placebo or probiotic interventions (Figure 11) in either the acne or nonacne populations.

**Figure 12:**
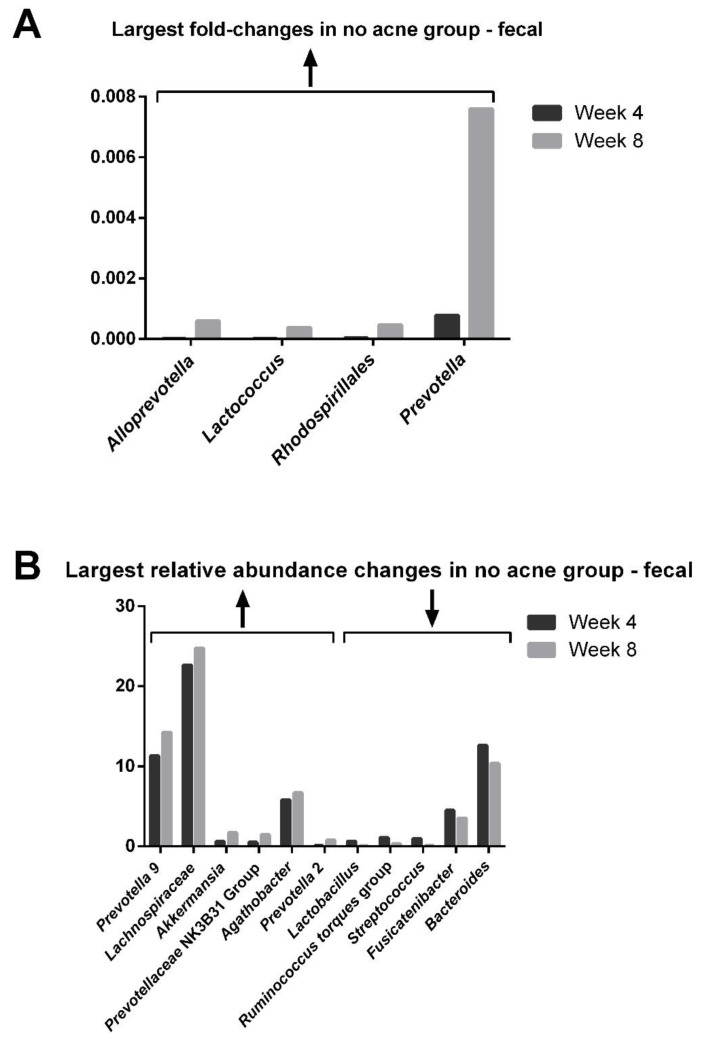
Change in the genera of the gut microbiome in those without acne. The fold-changes (**A**) and relative abundance (**B**) of the gut microbiome bacteria.

Despite the lack of shifts in overall diversity, several patterns emerged when stratifying the gut microbiome by those with and without acne. The largest bacterial changes in the fecal genera after 4 weeks on the probiotic in the no acne group are depicted in Figure 12 and include *Alloprevotella* (42-fold increase), *Lactococcus* (18-fold increase), *Rhodospirillales* (11-fold increase), and *Prevotella* (9.7-fold increase). The largest relative abundance changes were in *Akkermansia* (2.8-fold increase), *Prevotellacae* NK3B31 group (2.9-fold increase), *Lactobacillus* (13-fold decrease), *Ruminococcus torques* group (3.5-fold decrease), and *Streptococcus* (12-fold decrease).

**Figure 13:**
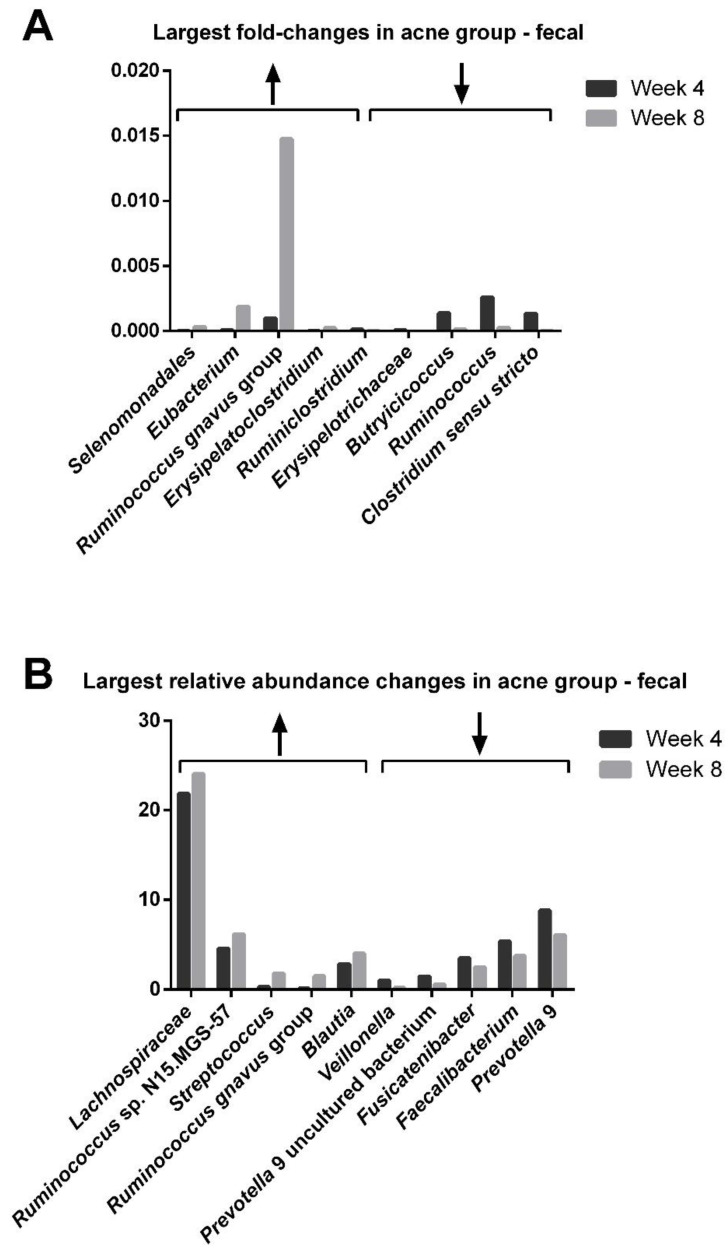
Change in the genera of the gut in those with acne. The fold-changes (**A**) and relative abundance (**B**) of the gut microbiome bacteria.

A similar analysis among those with acne (Figure 13) showed that the fecal bacterial genera with the largest bacterial changes after 4 weeks on the probiotic included *Selenomonadales* (16-fold increase), *Ruminococcus gnavus* group (15-fold increase), *Erysipelatodostridium* (13-fold increase), *Ruminidostridium* (7.0-fold decrease), *Erysipelotrichaceae* (9.0-fold decrease) *Butyricoccus* (8.6-fold decrease), *Ruminiococcus* (10-fold decrease), and *Clostridium sensu stricto* (34-fold decrease). The largest relative abundance changes were in *Streptococcus* (6.2-fold increase), *Ruminococcus gnavus group* (15-fold increase), and *Veilonella* (5.3-fold decrease).

### 3.5. Changes in the Blood Short-Chain Fatty Acids

**Figure 14:**
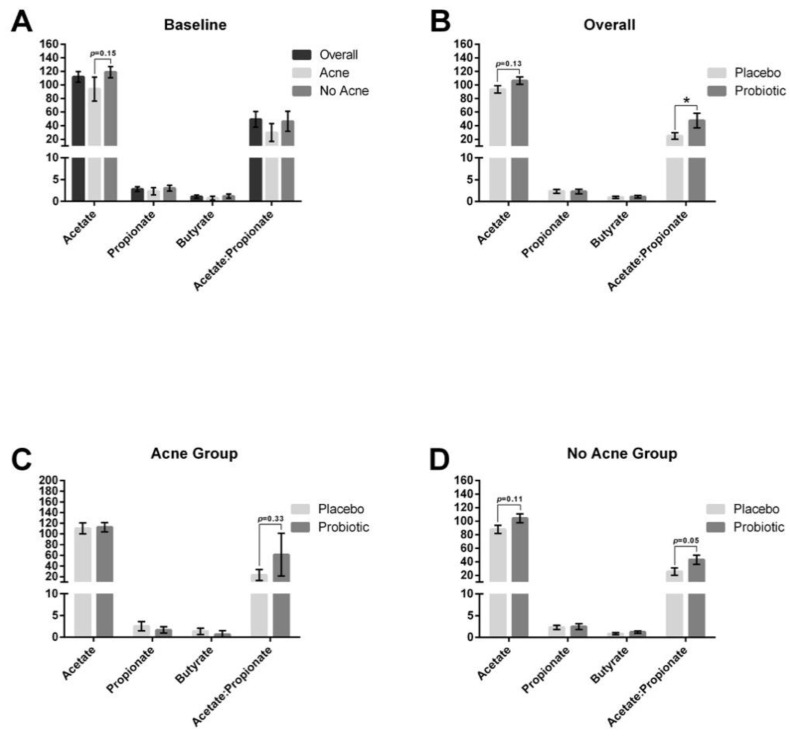
Shift in plasma short-chain fatty acids. (**A**) Short-chain fatty acids were measured at baseline for the entire study population (*n* = 25), acne subpopulation (*n* = 7), and no acne subpopulation (*n* = 18). There was a trend toward a decrease in acetate levels in those with acne (*p* = 0.15). (**B**) Probiotic supplementation led to an increasing trend in acetate levels (*p* = 0.13) and a significant increase in the acetate/propionate ratio in the overall population. (**C**) When stratifying for those with acne, probiotic supplementation led to a 2.6-fold increase in the acetate/propionate ratio that was not statistically significant (*p* = 0.33). (**D**) When stratifying for those without acne, probiotic supplementation led to an increasing trend in acetate levels (*p* = 0.11) and in the acetate/propionate ratio (*p* = 0.05). * *p* ≤ 0.05.

Prior to any supplementation, we noted that those with acne had a trend toward lower acetate levels (*p* = 0.15) without differences in butyrate and propionate levels in the blood (Figure 14). Probiotic supplementation led to an increasing trend in acetate levels (Figure 4B, *p* = 0.13) and a significant increase in the acetate/propionate ratio. Subanalysis of the no acne group showed increasing acetate levels approaching significance (Figure 4D, *p* = 0.11) and an increase in the acetate/propionate ratio (*p* = 0.05). Subanalysis of the acne group showed a 2.6-fold increase in the acetate/propionate ratio, but this difference was not statistically significant (*p* = 0.33).

## 4. Discussion

Our study shows that spore-based probiotic supplementation can modulate the skin’s biophysical properties and the sebum excretion rate. A gut–skin connection has long been postulated in other traditions such as traditional Chinese medicine, naturopathic medicine, and Ayurvedic medicine. Our work contributes clinical evidence for such a connection.

Acne-related clinical studies are typically carried out over 8 to 12 weeks, and our study was a pilot study with 4 weeks of supplementation. Nevertheless, our findings of a 37% reduction in the total lesion count at 4 weeks of supplementation are in agreement with another probiotic clinical study of acne that showed a 38% reduction in total lesion counts after 4 weeks [16]. Therefore, even though this study is pilot in nature, the improvement in acne, along with a trend toward a reduction in sebum, suggest that modulation of sebum may contribute to acne improvement. One of the advantages of our study is that each person served as their own control and received a placebo prior to probiotic supplementation to decrease interindividual differences with acne assessments.

The results indicated evidence for the presence of “leaky gut”, especially among those with acne. Although LPS did not have any changes in the overall population, LPS levels increased with placebo supplementation in the acne subpopulation, which normalized with probiotic supplementation. Furthermore, the FABP-2 marker was elevated at baseline for those with acne and had a reducing trend after probiotic supplementation in this group. FABP-2 is reported to be a marker of gut permeability [31], but it is additionally involved in fatty acid transport and lipid absorption [32], and high-fat diets may increase the levels of FABP-2 [33]. Therefore, it is possible that the elevated FABP-2 may represent a higher fat intake or a greater sensitivity to fat at the gut level of those with acne. Regardless, there was a trend toward reducing the FABP-2 levels after probiotic supplementation, suggesting that the probiotics may normalize or reduce FABP-2 levels in those with acne. This reduction in FABP-2 also correlates with a decrease in sebum production and with an improvement in clinical acne.

The LPS and FABP-2 results suggest that “leaky gut” may be present in those with acne and that probiotic exposure may normalize intestinal barrier function. However, when considering zonulin, a protein thought to also be reflective of intestinal permeability [34], the acne group did not demonstrate a trend toward elevated levels. Furthermore, probiotic exposure did not statistically shift zonulin levels in the acne group (*p* = 0.052). It is not clear why zonulin remained unchanged while LPS and FABP-2 shifted, but it may reflect their differential abilities in assessing the state of the gut barrier. It is important to note that while LPS, FABP-2, and zonulin are convenient measures that can be used to assess intestinal permeability, they are not considered the gold standard approach. Instead, future studies may consider measurements of the ratio of lactulose to mannitol excretion to assess intestinal permeability [35].

Several interesting shifts in the gut and skin microbiome were noted even in the absence of shifts in the overall Shannon diversity measures. The mix of five probiotic bacteria used in this study (*Bacillus* species) is composed of spore-forming bacteria that are typically abundant in the small intestine. Our sampling method was focused on fecal collection that is enriched for colonic and rectal organisms and may not directly reflect the state of the distal small intestine or the proximal large intestine. Regardless, bacterial shifts in the distal small intestine and proximal large intestine may still have “downstream” impacts that could be observed as significant shifts upon fecal collections.

Among the population without acne, supplementation with probiotics led to an increase in *Akkermansia*, in agreement with previous supplementation studies with *Bacillus*-based probiotics [36], along with an increase in *Lactococcus* and *Prevotella* abundance. Interestingly, *Lactococcus* may have an anti-inflammatory effect [37], while *Prevotella* is associated with a non-Western diet that is higher in complex fibers [38,39] and the production of short-chain fatty acids [40]. Both *Akkermansia* and *Prevotella* are known to produce short-chain fatty acids [40], which may be associated with the increasing trend in blood acetate levels and the acetate/propionate ratio after probiotic supplementation.

Among those with acne, probiotic supplementation increased in the presence of the *Lachnospiraceae* and *Ruminococcus gnavus* group and a decrease in the *Butyricicoccus* species. The *Lachnospiraceae* family of bacteria, which includes *Ruminococcus gnavus*, are short-chain fatty-acid-producing bacteria, especially acetate and propionate. This is especially notable in the acne group, which had a large effect size in the acetate/propionate ratio with a 2.6-fold increase, although it did not reach statistical significance. The acne subanalysis is underpowered due to only seven subjects for analysis, and our findings warrant future studies in a larger study population. While it is still not clear how the gut may communicate with the skin in those with acne, our findings suggest that short-chain fatty acids deserve further scrutiny.

The skin biophysical assessments showed no change in skin hydration and an increase in TEWL after supplementation with probiotics. However, there were no instances of dry or irritated skin that were noticed as a complaint or objectively noted among the participants. Therefore, TEWL may reflect that sebum is a contributor to skin barrier function since there was a trend toward a reduction in the sebum excretion rate.

This study had several limitations. This was a pilot study with a low number of subjects with acne. However, this study relied on validated measures such as total lesion count, and our findings were in agreement with a previous report of oral probiotic effects on acne [16]. Moreover, the lesion counts were associated with a decreasing trend in sebum production, further supporting our observations. A second limitation is that our fecal collections were more representative of the distal colonic microbiome rather than reflecting the entire GI tract. However, this is a common limitation in most gut microbiome analyses. To mitigate this limitation, we included plasma assessments for gut-derived markers and short-chain fatty acids so that we could make secondary assessments to augment our gut microbiome findings. A third limitation is that supplementation was only conducted for 4 weeks. Four weeks is typically sufficient to assess for changes in the gut, but changes in acne are better assessed over an 8- to 12-week period. Our study served as a pilot and was strengthened by the serial exposure to a placebo and then a probiotic. Therefore, each person served as their own control, and this increased the overall power of the study. Future studies with acne should build upon the pilot results here to conduct a study over an 8- or 12-week period. Finally, we did not place any restrictions on the diet of the participants, and this may have contributed to any variability in our results. However, we elected to perform this study in a real-world setting and directed people to eat as they regularly would. Moreover, each person served as their own control, which controls for the influence of diet and strengthens our findings.

In conclusion, spore-based probiotic supplementation led to shifts in the gut microbiome and a trend toward decreased sebum production, especially among those with acne. In those with acne, total, noninflammatory, and inflammatory lesion counts were improved after 4 weeks of probiotic supplementation. *Akkermansia* increased in the gut microbiome of those without acne, and *Lachnospiraceae* and *Ruminococcus gnavus* increased in the gut microbiome of those with acne. Probiotic supplementation increased the circulating acetate/propionate ratio. This study warrants further research into spore-based probiotics, and future studies will better delineate the role of oral probiotics in modulating short-chain fatty acids such as acetate, facial sebum production, and acne.

## Data Availability

No publicly archived datasets.

## References

[1] Dessinioti C., Katsambas A. (2017). Propionibacterium acnes and antimicrobial resistance in acne. Clin. Dermatol..

[2] Schwartz B.S., Graber C.J., Diep B.A., Basuino L., Perdreau-Remington F., Chambers H.F. (2009). Doxycycline, not minocycline, induces its own resistance in multidrug-resistant, community-associated methicillin-resistant *Staphylococcus aureus* clone USA300. Clin. Infect. Dis..

[3] Garrett J.P.-D., Margolis D.J. (2012). Impact of Long-Term Antibiotic Use for Acne on Bacterial Ecology and Health Outcomes: A Review of Observational Studies. Curr. Derm. Rep..

[4] Salem I., Ramser A., Isham N., Ghannoum M.A. (2018). The Gut Microbiome as a Major Regulator of the Gut-Skin Axis. Front. Microbiol..

[5] Zakostelska Z., Malkova J., Klimesova K., Rossmann P., Hornova M., Novosadova I., Stehlikova Z., Kostovcik M., Hudcovic T., Stepankova R. (2016). Intestinal Microbiota Promotes Psoriasis-Like Skin Inflammation by Enhancing Th17 Response. PLoS ONE.

[6] Thio H.B. (2018). The Microbiome in Psoriasis and Psoriatic Arthritis: The Skin Perspective. J. Rheumatol..

[7] Scher J.U., Ubeda C., Artacho A., Attur M., Isaac S., Reddy S.M., Marmon S., Neimann A., Brusca S., Patel T. (2015). Decreased bacterial diversity characterizes the altered gut microbiota in patients with psoriatic arthritis, resembling dysbiosis in inflammatory bowel disease. Arthritis Rheumatol..

[8] Yan H.M., Zhao H.J., Guo D.Y., Zhu P.Q., Zhang C.L., Jiang W. (2018). Gut microbiota alterations in moderate to severe acne vulgaris patients. J. Dermatol..

[9] Deng Y., Wang H., Zhou J., Mou Y., Wang G., Xiong X. (2018). Patients with Acne Vulgaris Have a Distinct Gut Microbiota in Comparison with Healthy Controls. Acta Derm.-Venereol..

[10] Nam J.H., Yun Y., Kim H.S., Kim H.N., Jung H.J., Chang Y., Ryu S., Shin H., Kim H.L., Kim W.S. (2018). Rosacea and its association with enteral microbiota in Korean females. Exp. Dermatol..

[11] Lee S.Y., Lee E., Park Y.M., Hong S.J. (2018). Microbiome in the Gut-Skin Axis in Atopic Dermatitis. Allergy Asthma Immunol. Res..

[12] Plaza-Diaz J., Ruiz-Ojeda F.J., Vilchez-Padial L.M., Gil A. (2017). Evidence of the Anti-Inflammatory Effects of Probiotics and Synbiotics in Intestinal Chronic Diseases. Nutrients.

[13] Liu Y., Alookaran J.J., Rhoads J.M. (2018). Probiotics in Autoimmune and Inflammatory Disorders. Nutrients.

[14] Hacini-Rachinel F., Gheit H., Le Luduec J.B., Dif F., Nancey S., Kaiserlian D. (2009). Oral probiotic control skin inflammation by acting on both effector and regulatory T cells. PLoS ONE.

[15] Notay M., Foolad N., Vaughn A.R., Sivamani R.K. (2017). Probiotics, Prebiotics, and Synbiotics for the Treatment and Prevention of Adult Dermatological Diseases. Am. J. Clin. Dermatol..

[16] Jung G.W., Tse J.E., Guiha I., Rao J. (2013). Prospective, randomized, open-label trial comparing the safety, efficacy, and tolerability of an acne treatment regimen with and without a probiotic supplement and minocycline in subjects with mild to moderate acne. J. Cutan. Med. Surg..

[17] Satoh T., Murata M., Iwabuchi N., Odamaki T., Wakabayashi H., Yamauchi K., Abe F., Xiao J.Z. (2015). Effect of Bifidobacterium breve B-3 on skin photoaging induced by chronic UV irradiation in mice. Benef. Microbes.

[18] Ishii Y., Sugimoto S., Izawa N., Sone T., Chiba K., Miyazaki K. (2014). Oral administration of Bifidobacterium breve attenuates UV-induced barrier perturbation and oxidative stress in hairless mice skin. Arch. Dermatol. Res..

[19] Mori N., Kano M., Masuoka N., Konno T., Suzuki Y., Miyazaki K., Ueki Y. (2016). Effect of probiotic and prebiotic fermented milk on skin and intestinal conditions in healthy young female students. Biosci. Microbiota Food Health.

[20] Fisk W.A., Lev-Tov H.A., Sivamani R.K. (2014). Botanical and phytochemical therapy of acne: A systematic review. Phytother. Res..

[21] Chilicka K., Rusztowicz M., Rogowska A.M., Szygula R., Asanova B., Nowicka D. (2022). Efficacy of Hydrogen Purification and Cosmetic Acids in the Treatment of Acne Vulgaris: A Preliminary Report. J. Clin. Med..

[22] Chilicka K., Rogowska A.M., Szygula R., Rusztowicz M., Nowicka D. (2022). Efficacy of Oxybrasion in the Treatment of Acne Vulgaris: A Preliminary Report. J. Clin. Med..

[23] Petukhova T.A., Foolad N., Prakash N., Shi V.Y., Sharon V.R., O’Brecht L., Ali I.A., Feldstein S., Halls J., Wang Q. (2016). Objective volumetric grading of postacne scarring. J. Am. Acad. Dermatol..

[24] Foolad N., Prakash N., Shi V.Y., Kamangar F., Wang Q., Li C.S., Sivamani R.K. (2016). The use of facial modeling and analysis to objectively quantify facial redness. J. Cosmet. Dermatol..

[25] Ornelas J., Rosamilia L., Larsen L., Foolad N., Wang Q., Li C.S., Sivamani R.K. (2016). Objective assessment of isotretinoin-associated cheilitis: Isotretinoin Cheilitis Grading Scale. J. Dermatol. Treat..

[26] Foolad N., Shi V.Y., Prakash N., Kamangar F., Sivamani R.K. (2015). The association of the sebum excretion rate with melasma, erythematotelangiectatic rosacea, and rhytides. Dermatol. Online J..

[27] Walters W., Hyde E.R., Berg-Lyons D., Ackermann G., Humphrey G., Parada A., Gilbert J.A., Jansson J.K., Caporaso J.G., Fuhrman J.A. (2016). Improved Bacterial 16S rRNA Gene (V4 and V4–5) and Fungal Internal Transcribed Spacer Marker Gene Primers for Microbial Community Surveys. mSystems.

[28] Caporaso J.G., Kuczynski J., Stombaugh J., Bittinger K., Bushman F.D., Costello E.K., Fierer N., Pena A.G., Goodrich J.K., Gordon J.I. (2010). QIIME allows analysis of high-throughput community sequencing data. Nat. Methods.

[29] Cole J.R., Wang Q., Cardenas E., Fish J., Chai B., Farris R.J., Kulam-Syed-Mohideen A.S., McGarrell D.M., Marsh T., Garrity G.M. (2009). The Ribosomal Database Project: Improved alignments and new tools for rRNA analysis. Nucleic Acids Res..

[30] Kumar S., Stecher G., Tamura K. (2016). MEGA7: Molecular Evolutionary Genetics Analysis Version 7.0 for Bigger Datasets. Mol. Biol. Evol..

[31] Stevens B.R., Goel R., Seungbum K., Richards E.M., Holbert R.C., Pepine C.J., Raizada M.K. (2018). Increased human intestinal barrier permeability plasma biomarkers zonulin and FABP2 correlated with plasma LPS and altered gut microbiome in anxiety or depression. Gut.

[32] Storch J., Corsico B. (2008). The emerging functions and mechanisms of mammalian fatty acid-binding proteins. Annu. Rev. Nutr..

[33] Lau E., Marques C., Pestana D., Santoalha M., Carvalho D., Freitas P., Calhau C. (2016). The role of I-FABP as a biomarker of intestinal barrier dysfunction driven by gut microbiota changes in obesity. Nutr. Metab..

[34] Fasano A. (2012). Zonulin, regulation of tight junctions, and autoimmune diseases. Ann. N. Y. Acad. Sci..

[35] Power N., Turpin W., Espin-Garcia O., Smith M.I., Consortium C.G.P.R., Croitoru K. (2021). Serum Zonulin Measured by Commercial Kit Fails to Correlate With Physiologic Measures of Altered Gut Permeability in First Degree Relatives of Crohn’s Disease Patients. Front. Physiol..

[36] Duysburgh C., Van den Abbeele P., Krishnan K., Bayne T.F., Marzorati M. (2019). A synbiotic concept containing spore-forming Bacillus strains and a prebiotic fiber blend consistently enhanced metabolic activity by modulation of the gut microbiome in vitro. Int. J. Pharm. X.

[37] Han K.J., Lee N.K., Park H., Paik H.D. (2015). Anticancer and Anti-Inflammatory Activity of Probiotic Lactococcus lactis NK34. J. Microbiol. Biotechnol..

[38] Ou J., Carbonero F., Zoetendal E.G., DeLany J.P., Wang M., Newton K., Gaskins H.R., O’Keefe S.J. (2013). Diet, microbiota, and microbial metabolites in colon cancer risk in rural Africans and African Americans. Am. J. Clin. Nutr..

[39] Yatsunenko T., Rey F.E., Manary M.J., Trehan I., Dominguez-Bello M.G., Contreras M., Magris M., Hidalgo G., Baldassano R.N., Anokhin A.P. (2012). Human gut microbiome viewed across age and geography. Nature.

[40] Koh A., De Vadder F., Kovatcheva-Datchary P., Backhed F. (2016). From Dietary Fiber to Host Physiology: Short-Chain Fatty Acids as Key Bacterial Metabolites. Cell.

